# A CAPS-based binding assay provides semi-quantitative validation of protein-DNA interactions

**DOI:** 10.1038/srep21030

**Published:** 2016-02-15

**Authors:** Yongyao Xie, Yaling Zhang, Xiucai Zhao, Yao-Guang Liu, Letian Chen

**Affiliations:** 1 State Key Laboratory for Conservation and Utilization of Subtropical Agro-Bioresources, South China Agricultural University, Guangzhou 510642, China; 2 Guangdong Provincial Key Laboratory of Protein Function and Regulation in Agricultural Organisms, South China Agricultural University, Guangzhou 510642, China; 3 Key Laboratory of Plant Functional Genomics and Biotechnology of Guangdong Provincial Higher Education Institutions, South China Agricultural University, Guangzhou 510642, China; 4 College of Life Sciences, South China Agricultural University, Guangzhou 510642, China

## Abstract

Investigation of protein-DNA interactions provides crucial information for understanding the mechanisms of gene regulation. Current methods for studying protein-DNA interactions, such as DNaseI footprinting or gel shift assays, involve labeling DNA with radioactive or fluorescent tags, making these methods costly, laborious, and potentially damaging to the environment. Here, we describe a novel cleaved amplified polymorphic sequence (CAPS)-based binding assay (CBA), which is a label-free method that can simplify the semi-quantitative validation of protein-DNA interactions. The CBA tests the interaction between a protein and its target DNA, based on the CAPS pattern produced due to differences in the accessibility of a restriction endonuclease site (intrinsic or artificial) in amplified DNA in the presence and absence of the protein of interest. Thus, the CBA can produce a semi-quantitative readout of the interaction strength based on the dose of the binding protein. We demonstrate the principle and feasibility of CBA using B3, MADS3 proteins and the corresponding RY or CArG-box containing DNAs.

With recent advances in functional genomics, examination of protein-DNA interactions has emerged as an increasingly important issue in the study of gene regulation, DNA replication, and recombination^1^. Large-scale screening of protein-DNA interactions may be conducted using yeast one-hybrid (Y1H) assays^2^, chromatin immunoprecipitation (ChIP) assays^3^ or DPI-ELISA^4^,^5^. Generally, these protein-DNA interactions require further validation by other methods such as DNaseI footprinting, electrophoretic mobility shift assays (EMSA), and DNA pull-down or immunoprecipitation assays^6^,^7^,^8^,^9^. DNaseI footprinting can determine the exact DNA sequence that a protein binds^6^, based on the observation that a bound protein protects the occupied nucleotides from digestion by DNaseI, which randomly digests the nucleotides not occupied by the interacting protein, resulting in a “footprint” on a gel. EMSA detects the protein-DNA interaction based on the observation that a protein-bound DNA complex generally migrates more slowly than the free DNA on a native gel^7^. Footprinting assays and EMSA usually use radioactive labels to detect the target DNA fragment, although some assays use fluorescent or biotin labels. Using radioactive isotopes makes these assays costly, requires special licensing, and is potentially unfriendly to users and the environment. Non-radioactive labeling also requires expensive reagents. Moreover, the quality of EMSA results depends on the strength of protein-DNA interactions. The labeled DNA may disassociate from the protein-DNA complex due to the ions in the buffer during electrophoresis on native gel resulting in a smeared pattern, especially in the case of weak protein-DNA interactions. Therefore, development of a simple, quantitative, low-cost method that enables rapid examination of protein-DNA interactions will facilitate functional genomics studies.

The cleaved amplified polymorphic sequence (CAPS) method is an extension of the restriction fragment length polymorphism (RFLP) method, using polymerase chain reaction (PCR) amplification for rapid genotyping^10^. The genetic differences between homologous DNA sequences can be revealed as simple fragment patterns after amplification, digestion with a specific restriction endonuclease, and resolution of the fragments by gel electrophoresis^10^. A restriction site in a *cis*-acting element can be used to study transcriptional regulation^11^,^12^. In 1983, Joachimiak *et al.* used an intrinsic 4-bp *Rsa* I site in the *trp* operator in the plasmid pRK9 to demonstrate trp repressor activity^11^. In a later study, an *Rsa*I site was introduced into the 4 degenerate nucleotides of the symmetrical fumarate and nitrate reductase (FNR)-recognition sequence (TTGATnnnnATCAA) to study the oxygen-dependent DNA binding of the FNR protein^12^. Based on these principles, with substantial modifications, we developed a simple CAPS-based binding assay (CBA) for the semi-quantitative validation of protein-DNA interactions without using radioactive labeling. The protein-DNA interaction can be simply determined by looking for RFLP fingerprinting in digested PCR-amplified DNA in the presence or absence of the protein, using standard gel electrophoresis. The interaction strength can be determined by the concentration of the binding protein in the assays.

## Results and Discussion

**Figure 1:**
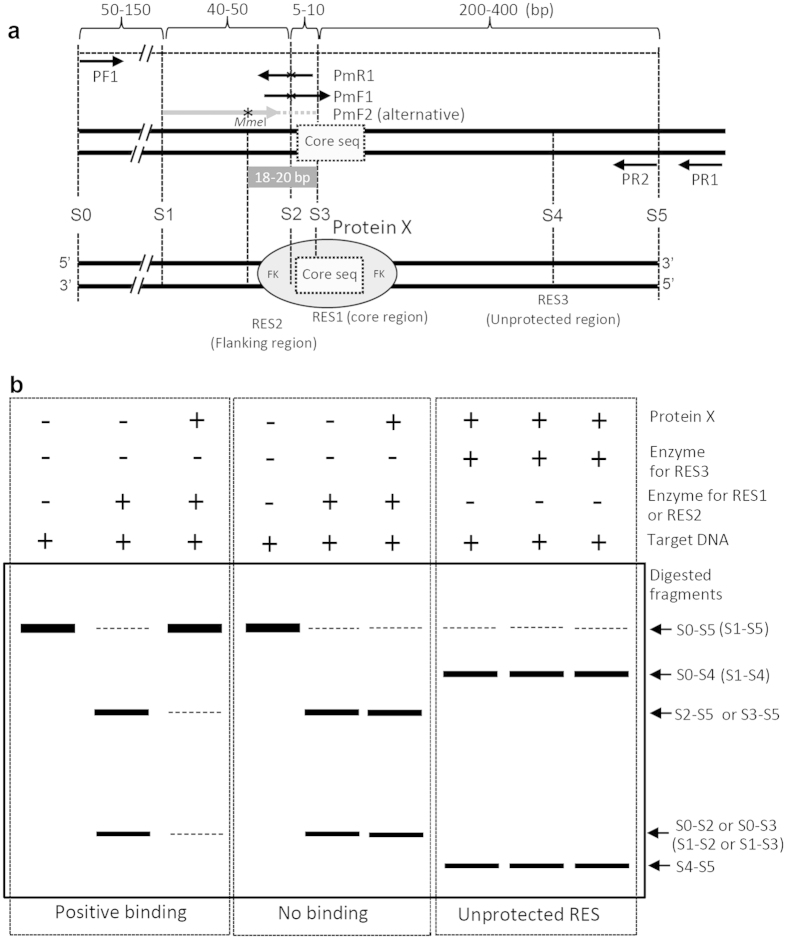
The principle of CBA for validation of protein-DNA interactions. **(a)** Diagram of CBA. The box bordered by the dotted line indicates the core sequence of the binding motif; the oval indicates the protein of interest (protein X) and the occupied region. RES1 and RES2 represent intrinsic or artificial restriction endonuclease sites in the core sequence or the flanking sequence (FK) of the binding motif on the target DNA. RES3 is outside of the region occupied by protein X. The target DNA fragment is prepared by nested-PCR: primers PF1/PR1 are used for the first PCR, the wild-type DNA fragment is produced by a second nested PCR with primers PF1/PR2 and the mutant DNA fragment is produced by overlapping PCR with two primer sets: PF1/PmR1 and PmF1/PR1 followed by the nested PCR amplification with PF1 and PR2. Alternatively, the PR2 may be combined with a long synthetic primer PmF2 (45–59 nt) in the second nested PCR. This long primer contains designed base mutation(s) (asterisk) to create an artificial recognition site for *Mme*I, whose cutting site is 20-bp away in the core sequence, thus simplifying the preparation of mutant DNA and reducing the risk of artifacts. **(b)** Expected CAPS patterns in CBA. If the RES1 or RES2 in the core or flanking sequence of the target DNA is protected from cleavage by the corresponding enzyme in the presence of protein X, it indicates that protein X binds the DNA (left). If protein X does not protect RES1 or RES2 from cleavage, this suggests that the protein does not interact with the DNA (middle). The cleavage of RES3 beyond the occupied region is not affected by mutation in the core sequence and the presence of protein X (right).

Figure 1 illustrates the principle of CBA: if a protein binds to its target motif in a DNA, a restriction endonuclease site (RES) in the core binding motif (core sequence) will be protected from digestion by the restriction endonuclease (Fig. 1a). Thus, the protein-DNA interaction can be simply determined by looking for a restriction fragment length polymorphism (RFLP) in PCR-amplified short DNA that was digested in the presence or absence of the protein (Fig. 1b). However, the availability of suitable intrinsic restriction sites in a given binding motif limits the application of this method. Generally, proteins bind DNA via a specific, conserved binding motif (core sequence), but the sequence region occupied by the protein can extend beyond the minimum core sequence. Therefore, nucleotides in the occupied flanking sequence (FK) of the core can be modified to create a unique, artificial RES without interfering with protein-DNA binding. Yet, the artificial RESs in the flanking sequences can be used for CBA because these sites are also protected from digestion by binding of the protein.

To perform CBA, the intrinsic or artificial RESs in a DNA fragment can be selected with the aid of commercial software such as DNAstar (http://www.dnastar.com/). The first criterion is that an intrinsic RES in the core sequence has priority, followed by an RES in the flanking sequence. If suitable intrinsic RESs do not occur in the core or flanking sequence, an artificial RES can be created by altering nucleotides either within the degenerate binding core sequence, or within the occupied flanking sequence, as long as the artificial RES does not affect binding of the protein. An artificial RES closer to the core sequence will produce more reliable results than a RES farther from the core. The second criterion is that choosing a restriction enzyme with fewer cutting sites will simplify the resulting cleavage pattern. To prepare wild-type and mutated DNA sequences for CBA, we recommend a (semi-)nested PCR procedure^13^, as illustrated in Fig. 1a. A pair of specific primers PF1/PR1 is used to amplify the template of the target DNA. If an intrinsic RES is available, the target DNA is obtained by a second nested PCR using primer pairs PF1/PR2; if an artificial RES is required, the mutated target DNA can be generated by overlapping PCR^14^ using three primer sets, PF1/PmR1, PmF1/PR1, and PF1/PR2, in succession. Alternatively, the second nested PCR can be conducted with PR2 and a long synthetic primer PmF2 (around 45–59 nt) containing the mutated nucleotide(s) for an artificial RES to simplify the preparation of a DNA mutant without using overlapping PCR. In this case, creation of an atypical type II restriction endonuclease recognition site of *Mme*I^15^ within the long primer is highly recommended. The cutting site of *Mme*I is 20 bp away from its recognition site; this may help avoid artifacts such as a false negative result due to interference with the interaction by the altered nucleotide(s) next to the core sequence. For artificial RESs that are close to the end of the fragment, polyacrylamide gel electrophoresis (PAGE) may be required to reveal the polymorphism in small digested products.

**Figure 2:**
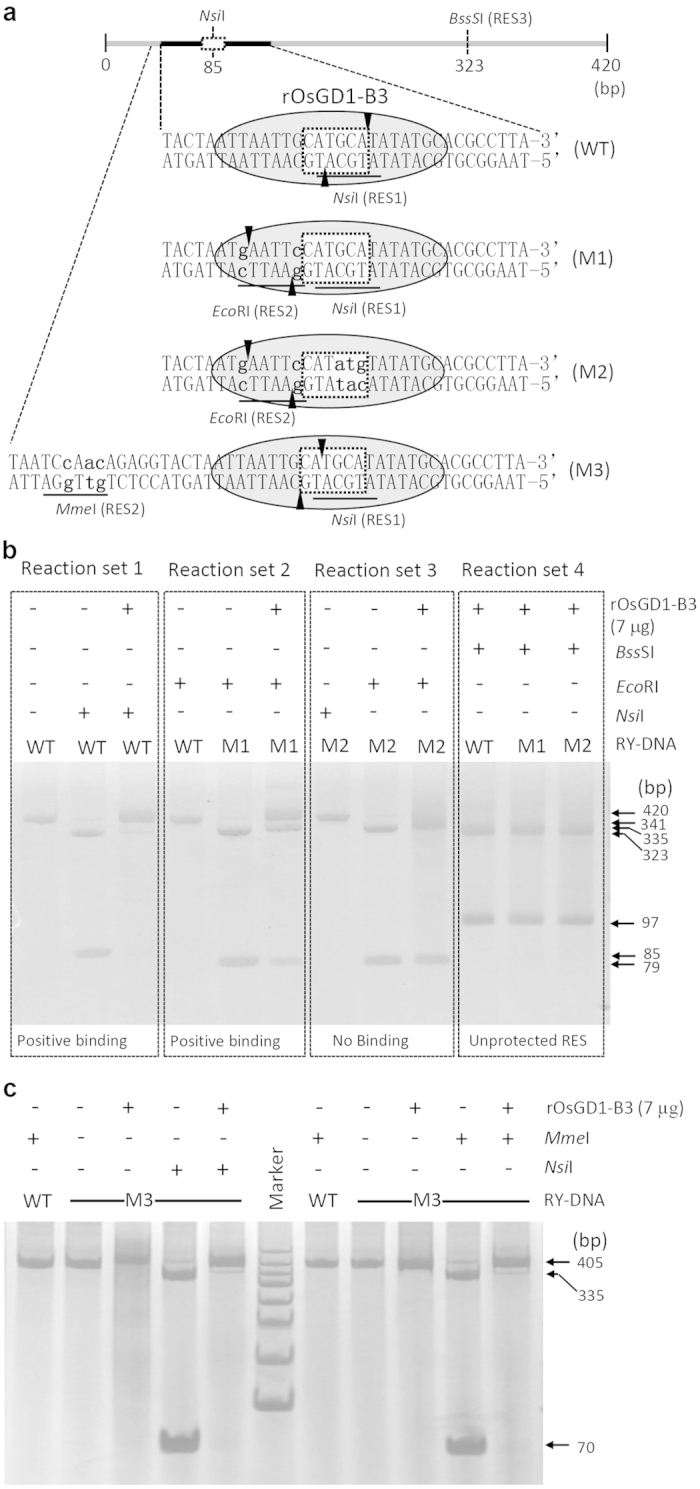
Validation of the interaction between OsGD1 and RY-DNA by CBA in different modes. **(a)** Diagram of the wild-type (WT) RY-DNA, the mutated DNAs (M1, M2, M3) and related RESs for CBA. The box bordered by the dotted line indicates the core sequence; the grey oval indicates the binding protein rOsGD1-B3 and the occupied region of the RY-DNA. *Nsi*I (RES1) is an intrinsic restriction enzyme site in the RY motif; *Eco*RI (RES2) is an artificial RES created by base changes (lower-case letters) in the flanking sequence in the mutated RY-DNA M1. M2 has additional mutations in the RY-motif. The *BssS*I RES is beyond the region occupied by the binding protein rOsGD1-B3. The mutated RY-DNA M3 contains a recognition site for *Mme*I and the *Mme*I cutting site is located in the core RY motif. The intrinsic *Nsi*I site in the RY motif remains intact but overlaps with the cutting site of *Mme*I. Vertical arrowheads indicate the cutting sites. (**b**) The CBA showed that the intrinsic *Nsi*I site in WT (Reaction set 1) and the artificial *Eco*RI site in M1 (Reaction set 2) were protected by binding of the rOsGD1-B3 protein. However, the artificial *Eco*RI in M2 was cut in the presence of rOsGD1-B3 protein (Reaction set 3), indicating that mutation in the flanking sequence does not interrupt M1-binding and protection of the artificial *Eco*RI RES in M1, but mutation in the core sequence abolishes protein binding and the protection of the RES. The *Bss*SI (RES3) beyond the protected region was not affected by mutation or the presence of rOsGD1-B3 (Reaction set 4). **(c)** The intrinsic RES *Nsi*I and the cutting site of *Mme*I in the M3 were protected by rOsGD1-B3 in CBA, indicating that the rOsGD1-B3 binds the M3 RY-DNA. Marker, 50 bp DNA ladder (TIANGEN BIOTECH, China).

In the present study, we used two DNA-protein combinations to demonstrate the feasibility of the CBA. In the first combination, a DNA fragment containing the RY (CATGCA) motif was used with two B3-domain proteins (OsGD1 and OsVAL2). The B3 domain of OsGD1 is known to bind the RY element of the *OsLFL1* promoter, as shown by EMSA^16^. To test these by CBA, we amplified a 420-bp RY-containing DNA fragment (RY-DNA) of the *OsLFL1* promoter (−1908 to −1488 bp) from the rice genome^17^. Sequence analysis showed that this fragment harbors 17 unique RESs, including two 6-bp RESs (*Nsi*I and *Sph*I) in the core sequence; these RESs are ideal for CBA (Figure S1). Therefore, we chose the *Nsi*I cutting site for CBA (Fig. 2a; Figure S1). The coding sequence for the B3 domain of OsGD1 was cloned into a prokaryotic expression vector and the 6× His-fused recombinant OsGD1-B3 (rOsGD1-B3) protein was purified from *E. coli* for *in vitro* binding assays (Figure S2). In the absence of rOsGD1-B3 protein, the wild-type RY-DNA fragment (W) was cut at the expected site by *Nsi*I, giving two products (Fig. 2b). In the presence of rOsGD1-B3 protein, the cleavage of RY-DNA by *Nsi*I was suppressed (Fig. 2b).

We also changed two nucleotides to create a unique artificial RES for *Eco*RI (RES2) at the 5′ flanking sequence of the RY motif, using overlapping-PCR with synthetic primers (Table S1) to generate an RY-DNA mutant named M1. This artificial *Eco*RI site was close to the core site, but did not affect the core sequence (Figure S1). CBA with the artificial RES2 (*Eco*RI) gave the same results as CBA with the intrinsic RES1 (*Nsi*I) (Fig. 2b). By contrast, if we used a fragment with a mutation in the core sequence of M1, the resultant M2 DNA with two mutations lost its protein-binding activity and the rOsGD1-B3 protein no longer protected the *Eco*RI site in the flanking sequence of M2 (Fig. 2b and Figure S1). However, the unrelated *BssS*I site (RES3), which is outside of the protected region, was not affected by the above mutations and rOsGD1-B3 protein (Fig. 2b and Figure S1).

To demonstrate the advantage of the use of an atypical type II restriction endonuclease *Mme*I in CBA, the recognition site of *Mme*I was integrated into a long synthetic primer. In the sequence generated with this primer, the mutated bases in the recognition site of *Mme*I are located 18-bp away, but the *Mme*I cutting site overlaps with the core sequence of the RY-motif (Fig. 1, S1 and Table S1). Our results demonstrated that both the intrinsic RES *Nsi*I and the cutting site of *Mme*I in the 405-bp M3 were protected by rOsGD1-B3 in CBA (Fig. 2a,c), confirming that rOsGD1-B3 binds the M3 RY-DNA. Since the altered nucleotides in the *Mme*I recognition site are far from (18–20 bp) the binding motif of the protein, the artificial *Mme*I recognition site likely does not affect the protein-DNA binding. Thus, application of *Mme*I (or other similar restriction enzymes) can have advantages compared to the use of typical type II restriction enzymes in CBA.

**Figure 3:**
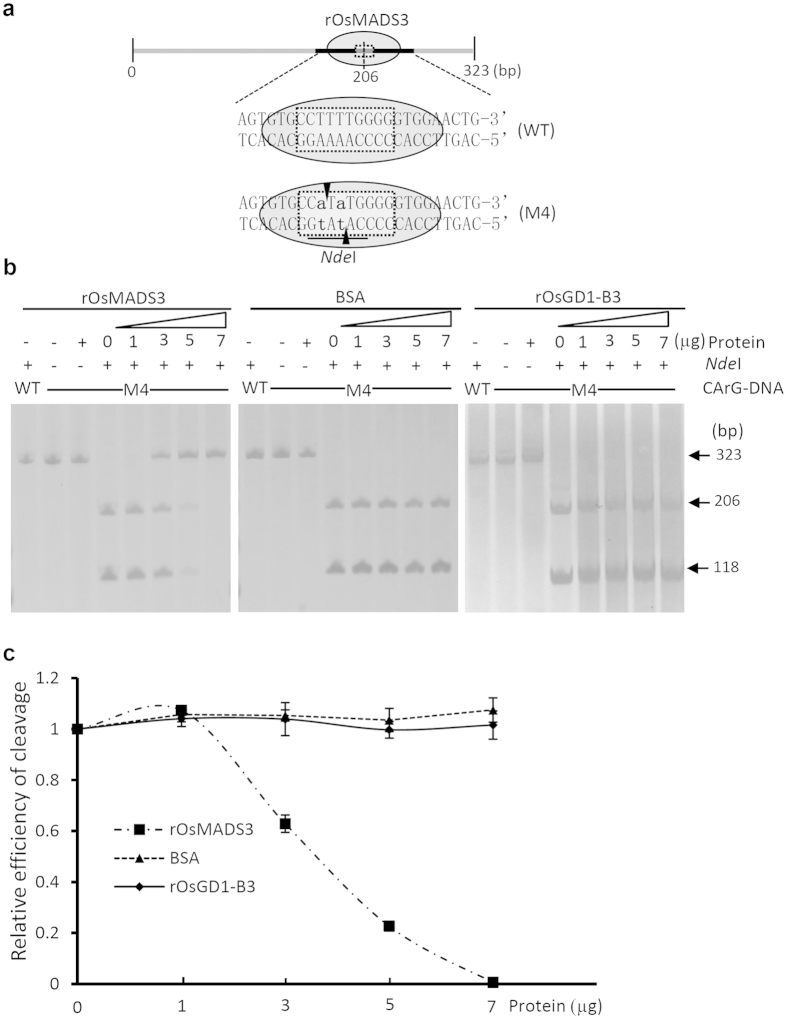
Validation of the interaction between OsMADS3 and CArG-DNA by CBA. **(a)** Diagram of the wild-type (WT) CArG-DNA, the mutated CArG-DNA M4 and RES used for CBA. The box bordered by the dotted line indicates the core sequence of the CArG-DNA; the grey oval indicates the binding protein rOsMADS3 and the occupied region on CArG-DNA. *Nde*I in M4 is an artificial RES created by changing two nucleotides of the degenerate core sequence [CC(A/T)_4_NNGG] of WT CArG-DNA. Vertical arrowheads indicate the cutting sites. **(b)** The WT CArG-DNA lacking the *Nde*I RES was resistant to *Nde*I digestion, while the M4 was cleaved by *Nde*I in the absence of rOsMADS3. The modified degenerate CArG-motif retained its protein-binding ability; thus the *Nde*I-digestion of CArG-DNA was suppressed by rOsMADS3 in a dose-dependent manner. The unrelated protein BSA or rOsGD1-B3 did not inhibit *Nde*I digestion, suggesting that the interactions revealed by CBA result from specific protein-DNA interactions. **(c**) Quantification of DNA cleavage in CBA. Intensity of the 118-bp band in each reaction containing 0, 1, 3, 5, 7 μg of testing proteins was quantified by Image J. Each value was normalized to the intensity of the band resulting from digestion without addition of binding protein (0 μg) to evaluate the relative efficiency of DNA cleavage by corresponding restriction enzyme in the CBA. Values are mean ± SEM, n = 3.

For the second DNA-protein combination, we used CBA to confirm the interaction between the transcription factor MADS3 and the CArG-box element in the promoter of metallothionein (*MT-1-4b*), an interaction that was previously shown by EMSA^18^. To confirm this combination by CBA, a 323-bp CArG-box containing fragment (CArG-DNA) was amplified from the rice genome with specific primers (Fig. 3, Figure S3 and Table S1). An artificial *Nde*I was introduced by changing two degenerate nucleotides in the core sequence [CC(A/T)_4_NNGG] of the CArG-DNA resulting in CArG-DNA mutant M4 (Fig. 3a and Figure S3). The wild-type CArG-DNA was resistant to *Nde*I, while the M4 DNA was cleaved by *Nde*I. In the presence of the binding protein rOsMADS3, DNA cleavage was inhibited and the inhibition efficiency increased with the amount of rOsMADS3 protein, indicating that rOsMADS3 binds specifically to the CArG-box of the *MT-1-4b* promoter (Fig. 3b). As a control for the specificity of protein-DNA binding in the CBA, we validated the interaction between CArG-DNA and the unrelated protein bovine serum albumin (BSA) or rOsGD1-B3 in different concentrations. The CBA results demonstrated that the addition of BSA or rOsGD1-B3 did not protect the CArG-DNA from digestion by *Nde*I (Fig. 3b). Moreover, the CBA also showed that the efficiency of DNA cleavage is protein dose-dependent for a specific protein-DNA interaction (Fig. 3b,c). Therefore, the interactions revealed by CBA result from a specific protein-DNA interaction.

Although, we could not exclude the possibility that an intact flanking sequence might be required for binding in certain cases, we tested our protein-DNA interactions with DNA pull-down assays^8^ using the wild-type and mutated DNA. The results showed that the M1 (mutated RY-DNA) and M4 (mutated CArG-DNA) with the artificial RES adjacent to the binding core sequence did not affect the interactions revealed by DNA pull-down assays in these two cases (Figure S4).

**Figure 4:**
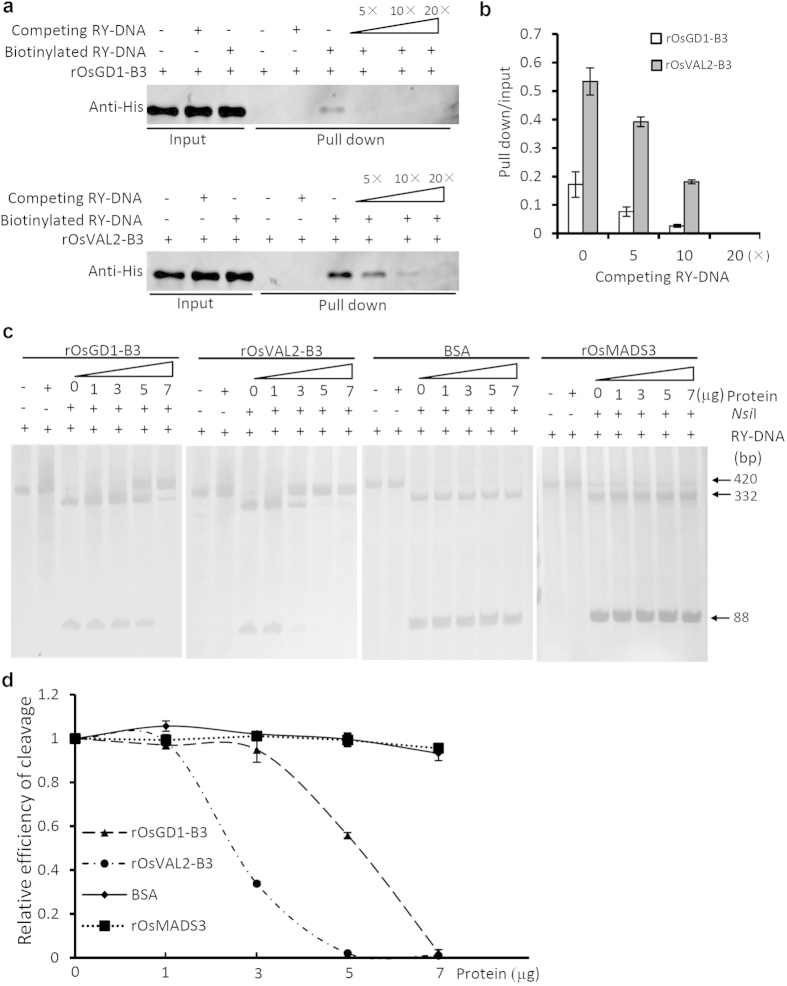
Quantification of the interaction strengths between B3 protein and RY-DNA by DNA pull-down assay and CBA **(a)** The interaction strengths of rOsGD1-B3 and rOsVAL2-B3 to the RY-DNA were assessed by DNA pull-down assays. In these assays, more rOsVAL2-B3 was pulled-down, compared with rOsGD1-B3 with equivalent amounts of biotinylated RY-DNA, as detected by western blotting with anti-His antibody against the His-tagged rOsGD1-B3 or rOsVAL2-B3. **(b)** To quantify the strength of protein-DNA interactions by pull-down assays, the signals from western blotting were normalized to the mean of inputs; three independent experiments were performed for the quantification. Values are mean ± SEM, n = 3. **(c)** Semi-quantitative evaluation of the interaction strengths by CBA. The DNA-binding B3 proteins suppressed the efficiency of *Nsi*I cleavage in a dose-dependent manner (the 1st and the 2nd panel). Moreover, addition of less rOsVAL2-B3 (3 μg) achieved a similar inhibitory effect on the RY-DNA cleavage as adding rOsGD1-B3 protein (5 μg) supporting that the rOsVAL2-B3 has stronger binding activity to the same RY-DNA than the rOsGD1-B3 *in vitro*. The unrelated proteins BSA and rOsMADS3 have no such inhibitory effect on the RY-DNA cleavage (the 3rd and the 4th panel). **(d)** Quantification of DNA cleavage in CBA. Intensity of the 88-bp band in each reaction containing 0, 1, 3, 5, 7 μg of binding proteins was quantified by Image J, respectively. ach value was normalized to the intensity of the band resulting from digestion without addition of binding protein (0 μg) to evaluate the relative efficiency of DNA cleavage by corresponding restriction enzyme in the CBA. Values are mean ± SEM, n = 3.

To evaluate the binding strength of different proteins to the same DNA *in vitro*, we chose another B3 protein, rOsVAL2-B3, for CBA^16^. We first tested the interaction strength between RY-DNA and rOsGD1-B3 or rOsVAL2-B3 using DNA-pull down assays^8^. The pull-down assay showed that rOsVAL2-B3 has a stronger interaction with RY-DNA than rOsGD1-B3 protein (Fig. 4a,b) *in vitro*. In CBA, the strength of the protein-DNA interaction has a negative correlation with the minimum dose of binding protein for DNA protection. Thus, a stronger protein-DNA interaction requires less protein to inhibit the cleavage of DNA by the restriction enzyme. Our CBA results demonstrated that inhibition of the RY-DNA cleavage requires 5 μg rOsGD1-B3 protein, but only 3 μg rOsVAL2-B3 protein is sufficient to achieve the same inhibitory effect (Fig. 4c). In contrast, the addition of the unrelated protein BSA or rOsMADS3 had no inhibitory effect on RY-DNA cleavage, indicating that BSA and rOsMADS3 do not bind the RY-DNA (Fig. 4c,d). Therefore, we can conclude that the interaction between the RY-DNA and rOsVAL2-B3 is stronger than that between the RY-DNA and rOsGD1-B3, which is consistent with the results of the DNA-pull down assays (Fig. 4a,b).

These CBA results confirmed that the DNA-binding proteins specifically bind *cis*-elements, thereby protecting the intrinsic or artificial RESs of the occupied region of the cognate DNA from cleavage by the corresponding restriction enzymes. The strength of protein-DNA binding may be reflected by the concentration of a given protein. CBA determines the interaction by fingerprinting a short, PCR-amplified DNA fragment based on protein-DNA binding, rather than separating the protein-bound DNA complex itself on a gel; therefore, the CAPS patterns provide a simple and clear-cut basis for drawing conclusions.

## Conclusion

Here we describe CBA, a simple, low-cost method that enables the rapid examination of protein-DNA interactions. Much as the development of CAPS analysis simplified genotyping, our CBA simplifies the validation of protein-DNA interactions by providing a label-free, CAPS-based method that can semi-quantitatively examine protein-DNA interactions. Basically, CBA requires a few micrograms (μg) of protein for an assay and the sensitivity of this method (estimated by minimum amount of protein) varies with the strength of protein-DNA interactions.

## Materials and Methods

### Expression and purification of B3 and MADS3 proteins

The coding sequences of the B3 domain of rice OsGD1 (Os07g0563300, amino acids 342–640) and OsVAL2 (Os07g0679700, amino acids 144–517) were amplified from rice cDNA and subcloned into the *pGoldI* expression vector (TAKARA, Japan) to generate the 6× his-tagged recombinant rOsGD1-B3 and rOsVAL2-B3 proteins. The recombinant proteins were extracted from *Escherichia coli* BL21 cells and purified by Ni-NTA resin (Qiagen, Germany) according to the manufacturer’s protocol. The MADS3 (Os01g0201700) coding sequence was subcloned into the pGEX4T-2 expression vector (GE Healthcare, USA) to generate the GST-tagged rOsMADS3 proteins. The rOsMADS3 protein was extracted from *E. coli* BL21 cells and purified by GSH-agarose (GE Healthcare, USA) according to the manufacturer’s protocol. To test the protein quality, the proteins were separated in SDS-PAGE gel and visualized by Coomassie Brilliant Blue staining and western blotting with corresponding antibodies (Figure S2).

### Preparation of wild-type and mutated DNAs for CBA

A 453-bp fragment (corresponding to the positions −1908 to −1455 bp from the start codon) of the *LFL* (Os01g0713600) promoter containing an RY element was amplified from rice genomic DNA using primers LFL-1F and LFL-1R (Table S1). As a template for the target wild-type RY-DNA, a semi-nested PCR was performed with LFL-1F and LFL-2R to produce a 420-bp (−1908 to −1488 bp) wild-type RY-DNA (WT) fragment (Figure S1 and Table S1). To generate the M1 and M2 mutants containing an artificial *Eco*RI restriction enzyme site (RES), two overlapping PCR^14^ amplifications were performed with primer pairs LFL-1F/M1-R and M1-F/LFL-1R or LFL-1F/M2-R and M2-F/LFL-1R in the first-round PCR, respectively. The primers LFL-1F/LFL-2R were used for the semi-nested PCR^13^. The resultant 420-bp mutant RY-DNA fragments M1 and M2 were then used for CBA. To prepare a 405-bp (−1893 to −1488 bp) mutated RY-DNA M3, LFL-2R and a 59-bp synthetic long primer M3-F containing a recognition site of *Mme*I were used for the second PCR (Figure S1 and Table S1). As a control, a 405-bp wild type RY-DNA was amplified with LFL-2F/LFL-2R (Figure S1 and Table S1).

A 371-bp fragment (−1112 to −741 bp) of the *MT-1-4b* (Os12g0571100) promoter containing a CArG-box element (CArG-DNA) was amplified from rice genomic DNA using primers MT-1F and MT-1R (Table S1) as template. The 323-bp (−1112 to −789 bp) wild-type CArG-DNA (WT) was amplified by semi-nested PCR using primers MT-1F/MT-2R (Table S1). An artificial *Nde*I RES was introduced into the CArG-box [CC(A/T)_4_NNGG] using two degenerate nucleotides to generate a CArG-DNA mutant M4 by overlapping-PCR amplification with primer pairs MT-1F/M4-R and M4-F/MT-1R in the first-round PCR, respectively. The primers MT-1F/MT-2R were used for the semi-nested PCR to generate the CArG-DNA mutant M4 harboring *Nde*I RES.

### Semi-quantitative analysis of protein-DNA interactions with CBA

For quantification with CBA, a suitable restriction endonuclease buffer was chosen for preparation of a mixture containing 150 ng target DNA, and 1 to 7 μg of protein in a final volume of 15 μl. After a pre-incubation at 37 °C for 30 min, 1 unit of restriction enzyme was added to the mixture and incubated for 1 hour at 37 °C for digestion. The denatured digested DNA mixture (5 μl) was loaded onto an 8% polyacrylamide gel for electrophoresis. The cleavage pattern was visualized by silver staining. The strength of protein-DNA interaction has a negative correlation with the dose of binding protein for DNA protection in CBA. Furthermore, one of the resultant bands (in small size) was used to evaluate the relative efficiency of DNA cleavage by the corresponding restriction enzyme. The intensity of each target band was normalized to that of the band digested without binding protein (0 μg). Mean and standard deviation were obtained from 3 independent replicates.

### Quantification of protein-DNA interaction with DNA pull-down assays

The DNA pull-down assay was performed according to a previous report^8^. The biotinylated DNAs were obtained by PCR amplification using synthetic biotinylated primers (Invitrogen Biotechnology Col Ltd, China). The proteins were pre-cleared with ImmunoPure streptavidin-agarose beads (Pierce, USA) for 1 hr, and then mixed with 1 μg of biotinylated DNA for 12 h in a microfuge tube on a rotating shaker. Due to the affinity of the streptavidin-agarose beads for biotinylated DNA, the DNA-protein complexes were pulled down with streptavidin-agarose beads by centrifugation at 200× *g* for 60 s. The pulled-down complex was washed 3 times with 1 ml ice-cold TBS buffer (20 mM Tris, 150 mM NaCl), separated on an SDS-polyacrylamide gel, and analyzed by western blotting. To quantify the strength of the DNA-protein complex, 5×, 10×, and 20× molar non-biotinylated DNAs were added to the pull-down mixture as competitors for the biotinylated DNA. The western blotting signals of the pulled-down proteins were quantified with ImageJ (National Institute of Health).

## Additional Information

**How to cite this article**: Xie, Y. *et al.* A CAPS-based binding assay provides semi-quantitative validation of protein-DNA interactions. *Sci. Rep.* **6**, 21030; doi: 10.1038/srep21030 (2016).

## Supplementary Material

Supplementary Information
