# Supplementary material for: A CAPS-based binding assay provides semi-quantitative validation of protein-DNA interactions
Source: Sci Rep. 2016 Feb 15;6:21030. doi: 10.1038/srep21030 (PMC4753479; doi:10.1038/srep21030)
Supplement: Supplementary Information [file srep21030-s1.pdf]

Supporting Information

A CAPS-based binding assay provides semi-quantitative validation of protein-DNA interactions

Yongyao Xie, Yaling Zhang, Xiucai Zhao, Yao-Guang Liu, Letian Chen*

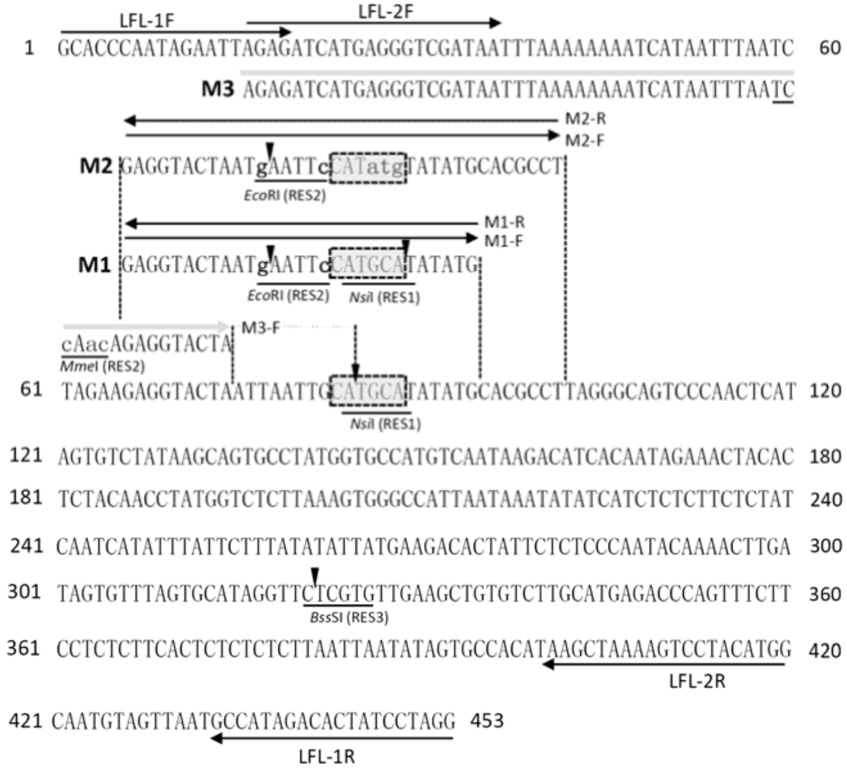

Figure S1

Figure S1. Primer design for preparation of wild-type and mutant RY-DNAs

The core sequence of the RY motif (CATGCA) is highlighted in a box bordered by a dashed line. The primers LFL-1F/LFL-1R were used for the first-round PCR. The 420-bp wild-type RY-DNA was generated by nested PCR with primers LFL-1F/LFL-2R. The primer sets LFL-1F/M1-R, M1-F/LFL-1R and primer sets LFL-1F/M2-R, M2-F/LFL-1R were used to create an artificial *Eco*RI site by altering specific nucleotides (lower-case letters) in M1 and M2 by overlapping PCR. The 405-bp M3 DNA was generated with long primer M3-F and LFL-2R, while the 405-bp wild-type DNA was amplified with LFL-2F/LFL-2R. The vertical arrowheads indicate the cutting sites.

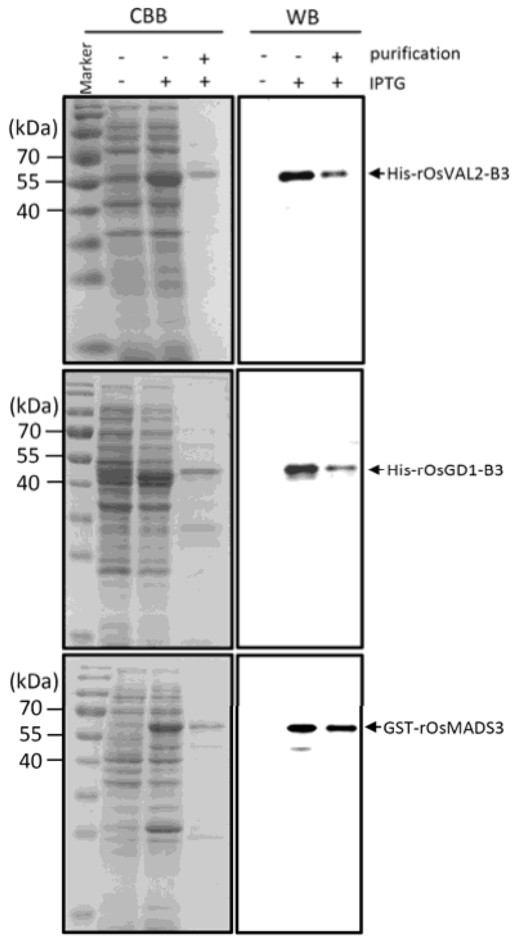

Figure S2

Figure S2. Quality of the recombinant proteins for CBA.

Each recombinant protein was expressed in *E. coli* and purified with specific resin according to the manufacturer's protocols. The lysates and purified proteins were examined by Coomassie Brilliant Blue (CBB) staining and western blotting (WB) with corresponding antibodies after SDS-PAGE. Marker, PageRuler™ Prestained Protein Ladder (10-180 kDa, Thermofisher Scientific, USA).

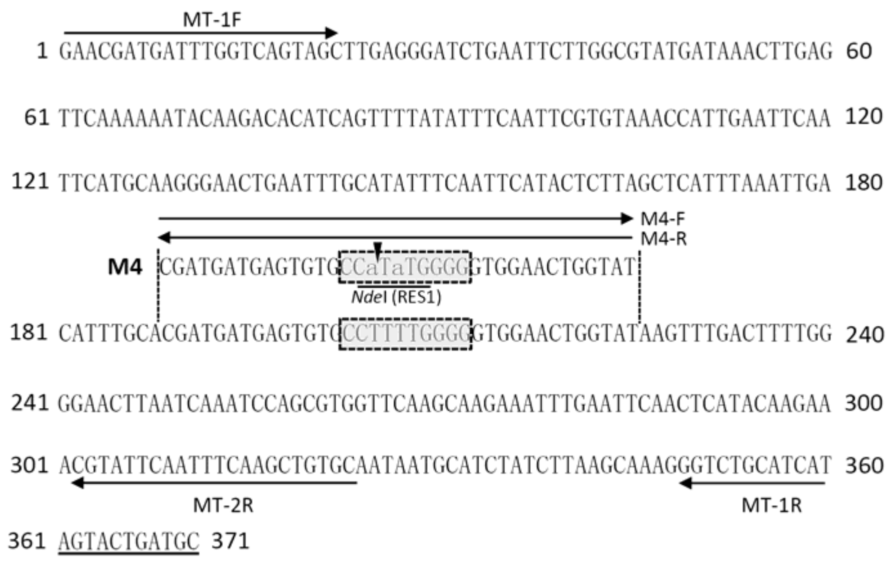

Figure S3

Figure S3. Primer design for preparation of wild-type and mutant CArG-DNAs

The degenerate core sequence of the CArG-box element [CC(A/T)$_4$NNGG] is highlighted in a box bordered by a dashed line. The primers MT-1F/MT-1R were used for the first round PCR and the 323-bp wild-type CArG-DNA was generated by nested PCR with primers MT-1F/MT-2R. The primer sets MT-1F/M4-R, M4-F/MT-1R were used to create an artificial *Nde*I site in the M4 mutant by overlapping PCR. The vertical arrowhead indicates the cutting site.

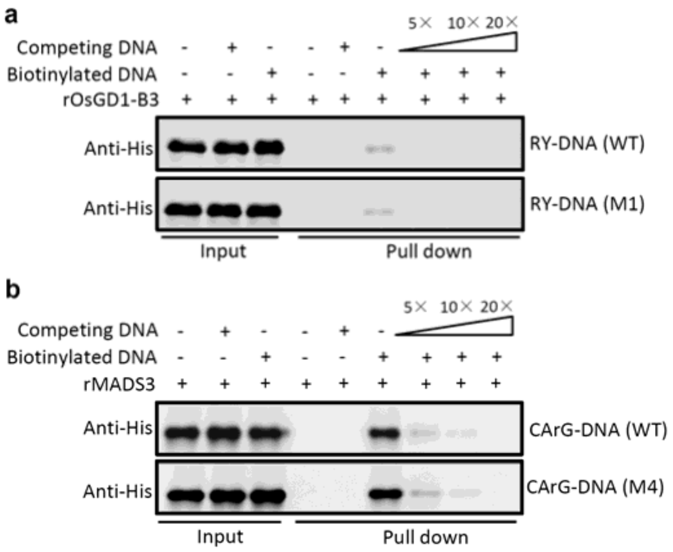

Figure S4

Figure S4. Comparison of DNA pull-down assays with wild type and mutant DNA containing an artificial RES.

(a) DNA pull-down assays were performed using wild-type RY-DNA and M1 DNA. The results showed that introduction of an artificial restriction enzyme site (RES) in the flanking sequence of core RY-motif may not significantly affect the protein-DNA interaction. **(b)** DNA pull-down assays were performed using wild-type CArG-DNA and M4 DNA. The results showed that creation of artificial RES using degenerate nucleotides in the core sequence of CArG-box may not affect the protein-DNA interaction.

Table S1. Primers used in this study

Primer	Sequence (5’--3’)	Purpose
LFL-1F	GCACCCAATAGAATTAGAG	420-bp WT DNA
LFL-2F	AGAGATCATGAGGGTCGATAA	405-bp WT DNA
LFL-1R	CCTAGGATAGTGTCTATGGC	All RY-DNA
LFL-2R	CCATGTAGGACTTTTAGCTT	All RY-DNA
M1-F	GAGGTACTAAT**g**AATT**c**CATGCATATATG	M1 DNA
M1-R	CATATATGCATG**g**AATT**c**ATTAGTACCTC	M1 DNA
M2-F	GAGGTACTAAT**g**AATT**c**CAT**atg**TATATGCACGCCT	M2 DNA
M2-R	AGGCGTGCATATA**cat**ATG**g**AATT**c**ATTAGTACCTC	M2 DNA
M3-F	AGAGATCATGAGGGTCGATAATTTAAAAAAAATCAT AATTTAATC**c**A**ac**AGAGGTACTA	M3 DNA
MT-1F	GAACGATGATTTGGTCAGTAGC	CArG-DNA
MT-1R	GCATCAGTACTATGATGCAGAC	CArG-DNA
MT-2R	GCACAGCTTGAAATTGAATACG	ACrG-DNA
M4-F	CGATGATGAGTGTGCC**a**T**a**TGGGGGTGGAACTGGTAT	M4 DNA
M4-R	ATACCAGTTCCACCCCCA**t**A**t**GGCACACTCATCATCG	M4 DNA
B-LFL-F	Biotin-TAATTGCATGCATATATGCACGC	WT DNA Pull-down assay
B-LFL-R	Biotin-GCGTGCATATATGCATGCAATTA	WT DNA Pull-down assay
C-LFL-F	TAATTGCATGCATATATGCACGC	Competing DNA
C-LFL-R	GCGTGCATATATGCATGCAATTA	Competing WT DNA
B-LFL-MF	Biotin-**g**AATT**c**CATGCATATATGCACGC	M1 DNA Pull-down assay
B-LFL-MR	Biotin-GCGTGCATATATGCATG**g**AATT**c**	M1 DNA Pull-down assay
C-LFL-MF	**g**AATT**c**CATGCATATATGCACGC	Competing M1 DNA
C-LFL-MR	GCGTGCATATATGCATG**g**AATT**c**	Competing M1 DNA
B-MT-F	Biotin-CGATGATGAGTGTGCCTTTTGGGGGTGGAACTGGTAT	WT DNA pull down assay
B-MT-R	Biotin-ATACCAGTTCCACCCCCAAAAGGCACACTCATCATCG	WT DNA pull down assay
MT-F	CGATGATGAGTGTGCCTTTTGGGGGTGGAACTGGTAT	Competing WT DNA
MT-R	ATACCAGTTCCACCCCCAAAAGGCACACTCATCATCG	Competing WT DNA
B-MT-MF	Biotin-CGATGATGAGTGTGCC**a**T**a**TGGGGGTGGAACTGGTAT	M4 DNA pull down assay
B-MT-MR	Biotin-ATACCAGTTCCACCCCCA**t**A**t**GGCACACTCATCATCG	M4 DNA pull down assay

Note: underlined nucleotides in lower case indicate the sites for mutagenesis.
